# Subcutaneous immunotherapy as a catalyst for decentralized, oncologist-led cancer care: a mixed-methods analysis

**DOI:** 10.1007/s12094-026-04393-7

**Published:** 2026-06-08

**Authors:** Eva Muñoz-Couselo, Julia Lostes, Giulia Pretelli, Manel Peiró, Joan Barrubés, Xavier Bayona

**Affiliations:** 1 Vall d’Hebrón Institute of Oncology (VHIO), Vall d’Hebrón Barcelona Hospital Campus, P. Vall d’Hebrón 119-129, 08035 Barcelona, Spain; 2 ESADE Business School, Institute for Healthcare Management, Universitat Ramon Llull, Barcelona, Spain; 3 Management Working Group of the Catalan Society of Family and Community Medicine & Bellvitge Primary Care Team. Primary Care Directorate of Costa de Ponent, Catalan Institute of Health, Barcelona, Spain

**Keywords:** Subcutaneous immunotherapy, Oncology care delivery, Decentralization, Patient-centered care, Health system sustainability, Immune checkpoint inhibitors

## Abstract

**Background:**

The widespread adoption of immune checkpoint inhibitors has significantly improved outcomes across multiple solid tumors but has also increased pressure on hospital-based oncology services, particularly oncology day hospitals. Subcutaneous (SC) formulations of immunotherapy offer a clinically validated alternative to intravenous (IV) administration and may facilitate more efficient, patient-centered models of cancer care delivery. However, the organizational, economic, and governance implications of this transition remain insufficiently explored.

**Methods:**

We conducted a mixed-methods analysis integrating four components: (1) a structured narrative review of clinical evidence regarding SC immune checkpoint inhibitors, including pharmacokinetics, efficacy, safety, and patient-reported outcomes; (2) an economic evaluation including cost-effectiveness and return-on-investment analyses; (3) benchmarking of national and international decentralized oncology care models; and (4) cross-sectional surveys of healthcare professionals (n = 84) and patients receiving immunotherapy (n = 76). Based on these findings, a hub-and-spoke care delivery framework supported by digital health tools was developed.

**Results:**

Across tumor types, SC immunotherapy demonstrated pharmacokinetic bioequivalence to IV formulations, with comparable efficacy and safety profiles. Treatment time was reduced by approximately 85–90%, resulting in improved patient-reported experience and reduced cumulative treatment burden. Adoption of SC immunotherapy was associated with up to 40% reduction in oncology day hospital occupancy and an estimated net annual saving of approximately €1,410 per patient. Both patients and healthcare professionals supported decentralized administration for selected clinically stable patients; however, acceptance was strongly dependent on preservation of oncologist-led care, strict patient selection, digital monitoring, and clearly defined escalation pathways.

**Conclusions:**

SC immunotherapy is a clinically effective and safe alternative to IV administration whose principal value lies in enabling selective decentralization of oncology care rather than simple logistical convenience. When implemented within a structured hub-and-spoke model that preserves medical oncology leadership, SC immunotherapy may improve patient experience, optimize healthcare resources, and strengthen long-term health system sustainability without compromising safety or continuity of care.

## Introduction

The introduction of immune checkpoint inhibitors (ICIs) has fundamentally transformed the treatment landscape of multiple solid tumors, leading to unprecedented improvements in survival and long-term disease control in cancers such as melanoma, non-small cell lung cancer, renal cell carcinoma, head and neck squamous cell carcinoma, and others. Over the past decade, immunotherapy has shifted cancer care from a predominantly acute, hospital-centered model toward a chronic disease paradigm, with a growing population of long-term survivors requiring sustained treatment and follow-up. While this therapeutic progress represents a major clinical success, it has also generated substantial organizational and operational challenges for oncology services [1, 10, 14–19].

In most healthcare systems, including those in Europe, immunotherapy delivery remains largely centralized within hospital-based oncology day units. This hospital-centric model was originally designed to manage the complexity and toxicity profiles of intravenous anticancer treatments, ensuring patient safety through close monitoring and specialized infrastructure. However, the rapid expansion of immunotherapy indications, together with increasing treatment duration and patient volumes, has led to progressive saturation of oncology day hospitals. Prolonged waiting times, limited capacity for new treatments, and increasing pressure on healthcare professionals have become common challenges, raising concerns regarding the sustainability of current care delivery models [1, 10, 47].

From the patient perspective, the burden associated with repeated hospital visits for intravenous treatment administration is increasingly recognized as a relevant component of treatment-related impact. Regular travel to tertiary centers, long chair times, and disruptions to daily life can negatively affect quality of life, work productivity, and psychosocial well-being, particularly for patients receiving long-term immunotherapy. As patient-reported outcomes and experiences gain importance in contemporary oncology practice, there is growing interest in care models that maintain clinical efficacy while reducing treatment burden and improving convenience [1, 4–6, 10, 11, 36, 46].

In this context, the development of subcutaneous (SC) formulations of immune checkpoint inhibitors represents a relevant technological and clinical innovation. Advances in pharmaceutical formulation, including the use of recombinant human hyaluronidase, have enabled the safe and effective subcutaneous administration of monoclonal antibodies that were traditionally delivered intravenously. Clinical trials across multiple tumor types have consistently demonstrated pharmacokinetic bioequivalence between SC and intravenous formulations, with comparable efficacy and safety profiles. Importantly, SC administration significantly reduces treatment time, often from 30–60 min of infusion to only a few minutes, without compromising clinical outcomes [1, 8–13, 31, 32, 37, 38].

Beyond pharmacological considerations, subcutaneous immunotherapy introduces the possibility of rethinking how and where cancer treatments are delivered. Shorter administration times and simplified logistics may facilitate treatment delivery outside traditional hospital-based oncology day units, including in community hospitals, outpatient clinics, or alternative care settings closer to patients’ homes. This shift aligns with broader healthcare trends toward decentralization, integration of digital health tools, and value-based care, aiming to improve efficiency while maintaining high standards of quality and safety [1, 10–12, 23, 31, 32, 37, 38, 41, 44–46, 51].

Several healthcare systems have already explored decentralized oncology care models, including hub-and-spoke networks, shared-care approaches, and home-based treatment programs for selected therapies. These initiatives have shown that, when appropriately coordinated and supported by clear clinical governance structures, decentralized care can improve accessibility and patient experience without compromising outcomes. However, most existing models have focused on chemotherapy, supportive care, or oral anticancer agents. The potential role of subcutaneous immunotherapy as a driver of decentralized oncology care has not yet been comprehensively evaluated [1, 4, 5, 10–12, 23, 31, 32, 37, 38, 40, 41, 43–46].

In addition to organizational considerations, the economic implications of immunotherapy delivery models are increasingly relevant. Immunotherapy represents a significant proportion of oncology drug expenditure, and its associated administration costs—including staff time, infrastructure use, and opportunity costs related to capacity constraints—are substantial. While drug acquisition costs are similar between SC and intravenous formulations, differences in administration time and resource utilization may translate into meaningful economic impacts at the system level. Understanding these implications is essential for healthcare planners and decision-makers aiming to ensure long-term sustainability [1, 4–6, 10, 31, 32, 37, 38, 47].

Another critical aspect of care redesign is the preservation of clinical leadership and coordination. As treatment delivery potentially moves beyond specialized oncology units, there is a risk of fragmentation of care or dilution of responsibility for toxicity monitoring and clinical decision-making. Medical oncology plays a central role in treatment selection, management of immune-related adverse events, and longitudinal patient follow-up. Any decentralized model involving subcutaneous immunotherapy must ensure that medical oncology retains clinical leadership and oversight, supported by standardized protocols, training, and digital tools that enable safe and coordinated care [1, 10, 23, 31, 32, 37, 38, 41, 44, 45, 54].

Despite growing interest in subcutaneous immunotherapy, the literature has largely focused on pharmacokinetics, efficacy, and patient preference, with limited integration of organizational, economic, and governance perspectives. There remains a gap in comprehensive analyses that examine subcutaneous immunotherapy not only as an alternative route of administration, but as a potential catalyst for broader transformation of oncological care delivery [1, 10, 31, 32, 37, 38].

The aim of this study is to address this gap by providing an integrated clinical, organizational, and economic analysis of subcutaneous immunotherapy as a driver of decentralized, patient-centered oncology care. By combining evidence from the literature, economic evaluation, stakeholder surveys, and benchmarking of national and international experiences, we propose a pragmatic care delivery model that leverages the advantages of subcutaneous immunotherapy while ensuring clinical safety, efficiency, and sustainability. Ultimately, this work seeks to inform clinicians, healthcare leaders, and policymakers on how subcutaneous immunotherapy may contribute to the evolution of oncology care in the immunotherapy era [1, 10, 23, 31, 32, 37, 38, 41, 44–47].

## Methods

### Study design

This study used a mixed-methods descriptive and analytical design to evaluate the clinical, organizational, and economic implications of subcutaneous (SC) immunotherapy as a potential driver of decentralized oncology care. The study integrated four complementary components: [1] a structured narrative review of clinical evidence, (2) an economic evaluation including cost-effectiveness and return-on-investment analyses, (3) cross-sectional stakeholder surveys involving healthcare professionals and patients, and (4) benchmarking of existing national and international decentralized oncology care models. Findings from these components were subsequently used to develop a pragmatic hub-and-spoke care delivery framework.

This mixed-methods approach was selected to combine clinical evidence with real-world operational, economic, and stakeholder perspectives, allowing a comprehensive evaluation of the feasibility and sustainability of decentralized SC immunotherapy delivery.

### Structured narrative review

A structured narrative review was conducted to identify evidence regarding the efficacy, safety, pharmacokinetics, patient-reported outcomes, and organizational implications of SC immune checkpoint inhibitors.

The literature search was performed in PubMed/MEDLINE, Embase, and the Cochrane Library for studies published between January 2018 and March 2025. Search terms included combinations of the following keywords and MeSH terms: “subcutaneous immunotherapy,” “subcutaneous checkpoint inhibitor,” “immune checkpoint inhibitors,” “pembrolizumab,” “nivolumab,” “atezolizumab,” “oncology care delivery,” “decentralized care,” “patient-reported outcomes,” and “hub-and-spoke model.”

Eligible studies included phase I–III clinical trials, regulatory reports, real-world observational studies, systematic reviews, and high-quality health services research publications relevant to SC immunotherapy delivery. Conference abstracts from major international oncology meetings (ASCO, ESMO, SITC) were also reviewed when full publications were not yet available.

Studies exclusively focused on non-oncology biologics or without specific relevance to SC immune checkpoint inhibitors were excluded. Study selection was performed independently by two investigators, with disagreements resolved by consensus.

Given the exploratory objective of the study, formal meta-analysis was not performed. Evidence was synthesized narratively according to four predefined domains: pharmacokinetic equivalence, efficacy and safety, patient-reported outcomes, and organizational implications for care delivery.

### Economic evaluation

An economic evaluation was conducted from the perspective of the public healthcare system using two complementary approaches: cost-effectiveness analysis (CEA) and return on investment (ROI) analysis.

### Cost-effectiveness analysis

Incremental cost-effectiveness ratios (ICERs) were estimated comparing SC versus intravenous (IV) immunotherapy administration. Quality-adjusted life years (QALYs) were derived from published patient-reported outcome data and validated quality-of-life instruments, including EORTC QLQ-C30-based utility estimates.

As clinical efficacy and survival outcomes were considered equivalent between SC and IV formulations based on available phase III data, differences in utility were primarily attributed to treatment convenience, reduced hospital time, and improved patient experience.

A willingness-to-pay threshold aligned with commonly accepted European health economic standards was applied.

### Return on investment analysis

ROI analysis evaluated direct and indirect administration-related costs, including nursing time, chair occupancy in oncology day hospitals, infusion infrastructure use, consumables, and opportunity costs associated with limited capacity.

A one-year time horizon was used for the base-case analysis. Scenario analyses were performed using alternative assumptions regarding treatment volume, adoption rate, and long-term operational impact.

Drug acquisition costs were assumed to be equivalent between SC and IV formulations according to current pricing structures and were therefore excluded as a differentiating variable.

Sensitivity analyses were performed using conservative and optimistic scenarios to test the robustness of the economic estimates.

### Stakeholder surveys

Two cross-sectional surveys were conducted between January and March 2025 to assess perceptions regarding SC immunotherapy and decentralized oncology care.

### Healthcare professionals

A structured electronic questionnaire was distributed to 84 professionals involved in cancer care, including medical oncologists, oncology nurses, hospital pharmacists, healthcare managers, primary care physicians, and pharmaceutical industry representatives.

The questionnaire explored perceived benefits and barriers of SC immunotherapy, safety concerns regarding decentralization, views on medical oncology leadership, and organizational implications.

### Patients

A separate questionnaire was administered to 76 adult patients receiving immunotherapy at a tertiary cancer center. Eligible patients were those receiving active immune checkpoint inhibitor treatment for solid tumors and able to provide informed consent.

Survey domains included treatment burden, hospital time, route-of-administration preferences, willingness to receive treatment closer to home, and perceptions regarding safety and continuity of oncologist-led care.

Questionnaires were developed specifically for this study based on literature review and expert consensus and were piloted in a small sample prior to implementation to improve clarity and content validity.

Responses were analyzed descriptively using frequencies and proportions. Subgroup analyses were performed according to professional role, treatment duration, and prior treatment experience when appropriate.

### Benchmarking of decentralized oncology care models

A benchmarking exercise was performed to identify national and international experiences relevant to decentralized oncology care delivery.

Programs from Spain, the United Kingdom, Australia, the Netherlands, and North America were included based on relevance to shared-care oncology models, hub-and-spoke systems, home-based anticancer treatment, or digitally supported outpatient oncology follow-up.

Each model was evaluated using a predefined framework including governance structure, role of medical oncology, patient selection criteria, digital integration, escalation pathways, safety monitoring, and reported implementation barriers.

The objective was not comparative effectiveness assessment but identification of transferable organizational principles and implementation strategies applicable to SC immunotherapy.

### Development of the proposed care delivery model

Based on the integrated findings from the literature review, economic evaluation, stakeholder surveys, and benchmarking analysis, a decentralized hub-and-spoke model for SC immunotherapy delivery was developed.

The model was designed around five core principles: preservation of medical oncology leadership, standardized patient selection criteria, protocolized toxicity monitoring, digital health integration, and clearly defined escalation pathways for immune-related adverse events.

Patient eligibility for decentralized administration was restricted to clinically stable patients with good performance status (ECOG 0–1), no recent grade ≥ 2 immune-related toxicity, and no immediate need for radiological or complex clinical reassessment.

The proposed framework was intended as an adaptable model for implementation across different healthcare systems while maintaining core standards of safety, quality, and patient-centered care.

### Clinical evidence supporting subcutaneous immunotherapy

#### Pharmacokinetic equivalence and regulatory validation

Across pivotal clinical development programs, subcutaneous (SC) formulations of immune checkpoint inhibitors consistently demonstrated pharmacokinetic bioequivalence to intravenous (IV) administration, fulfilling predefined regulatory criteria for area under the curve (AUC), trough concentrations, and systemic exposure consistency.

Phase III studies evaluating SC formulations of pembrolizumab, nivolumab, and atezolizumab showed that SC administration achieved non-inferior exposure profiles compared with IV delivery, without clinically meaningful differences in peak plasma concentrations. These findings supported regulatory approval of SC formulations in multiple jurisdictions and established SC administration as a validated therapeutic alternative rather than an experimental route of delivery [1, 7, 24–26, 31, 32, 37, 38].

The incorporation of recombinant human hyaluronidase enabled administration of high-concentration monoclonal antibodies in reduced injection volumes with predictable absorption kinetics and minimal local toxicity. Importantly, no evidence of altered immune activation or increased acute toxicity related to absorption dynamics was observed across studies [31, 32, 37–40].

These findings provide the pharmacological foundation required to support broader organizational redesign based on SC immunotherapy delivery.

### Efficacy across tumor types

Across multiple solid tumors, including melanoma, non-small cell lung cancer, renal cell carcinoma, and other advanced malignancies, SC immunotherapy demonstrated efficacy outcomes comparable to IV administration.

Objective response rates, progression-free survival, and overall survival were consistently non-inferior between SC and IV formulations in available phase II–III studies. This was observed for SC pembrolizumab, nivolumab, and atezolizumab across both early and metastatic treatment settings [2, 3, 7, 14–19, 25, 26, 29, 31, 32, 37, 38].

These findings are particularly relevant in the context of long-term immunotherapy, where durable disease control is a major therapeutic objective. Available follow-up data showed sustained responses over time without evidence of compromised long-term efficacy, supporting the use of SC administration not only during induction phases but also during prolonged maintenance treatment.

Additionally, extended-interval dosing strategies (such as pembrolizumab every 6 weeks and nivolumab every 4 weeks) further reinforce the operational advantages of SC administration by reducing treatment frequency while maintaining clinical outcomes, thereby maximizing efficiency in oncology day units and facilitating decentralized care models [27, 38].

### Safety, tolerability, and patient selection for decentralized delivery

The safety profiles of SC and IV immunotherapy formulations were broadly comparable, with no new or unexpected immune-related adverse events observed with SC administration.

Rates of grade 3–4 immune-related toxicity remained consistent with the established safety profile of immune checkpoint inhibitors and did not differ significantly between routes of administration. Injection-site reactions were the most common route-specific adverse events associated with SC delivery; however, these were typically mild, transient, and self-limited, rarely leading to treatment discontinuation. In contrast, infusion-related reactions were less frequent compared with IV administration [1, 10–21, 31, 32, 37, 38, 52–54].

Importantly, safe decentralization requires strict patient selection rather than universal eligibility. Patients considered most appropriate for decentralized SC administration were those with stable disease, good functional status (ECOG 0–1), no recent grade ≥ 2 immune-related adverse events, and no immediate need for radiological reassessment or complex therapeutic decision-making.

Patients with active toxicity, clinical deterioration, poor performance status, or uncertain treatment indication should remain under direct hospital-based oncology supervision. This distinction is critical to ensure that decentralization improves convenience without compromising safety or delaying recognition of immune-related toxicity, in line with European Society for Medical Oncology recommendations for immunotherapy toxicity management [20, 21, 54].

These findings support decentralization as a selective, protocol-driven strategy rather than a generalized transfer of care.

### Patient-reported outcomes and treatment experience

Patient-reported outcomes emerged as one of the most clinically meaningful differentiators between SC and IV immunotherapy.

Across multiple studies, patients receiving SC formulations consistently reported improved treatment experience, primarily driven by shorter administration times, reduced time spent in hospital, decreased travel burden, and greater flexibility in daily life. Treatment duration was reduced by approximately 85–90% compared with IV infusion, significantly lowering cumulative treatment burden [1, 6, 10–21, 31, 32, 36–38, 46].

Improvements in global quality-of-life scores measured using validated instruments such as EORTC QLQ-C30 exceeded thresholds considered clinically meaningful. Patient preference studies consistently showed a strong preference for SC administration, with most patients expressing willingness to continue treatment via the SC route when given the choice.

However, patient preference was not driven exclusively by convenience. Patients also emphasized the importance of maintaining continuity with their treating medical oncologist and expressed greater acceptance of decentralized administration when clear specialist supervision and rapid escalation pathways were guaranteed.

This finding reinforces that patient-centered care requires both convenience and preserved oncologist-led clinical oversight.

### Implications for clinical practice

Taken together, the available clinical evidence establishes SC immunotherapy as a therapeutically equivalent alternative to IV administration, combining maintained efficacy, comparable safety, and improved patient experience.

Importantly, these advantages are achieved without reducing therapeutic complexity. SC immunotherapy does not modify the biological mechanism of immune checkpoint inhibition, and patients continue to require specialized oncological assessment, toxicity monitoring, and complex therapeutic decision-making throughout treatment.

The principal innovation therefore lies not in simplification of cancer care, but in simplification of drug delivery logistics. This distinction is essential: decentralization should not imply de-medicalization.

When implemented within a structured hub-and-spoke model, with clear medical oncology leadership, standardized protocols, digital monitoring, and defined escalation pathways, SC immunotherapy provides a robust clinical foundation for decentralized, patient-centered oncology care that maintains safety, quality, and continuity of treatment [1, 10, 23, 30–34, 37, 38, 41, 44].

### Organizational impact and decentralized care delivery model

#### Impact on oncology day hospital capacity

The rapid expansion of immune checkpoint inhibitor use has substantially increased demand for oncology day hospital resources, particularly in high-volume tertiary cancer centers. Intravenous (IV) administration requires prolonged chair occupancy, nursing supervision, infusion preparation, and dedicated infrastructure, contributing to capacity constraints, longer waiting times, and reduced flexibility for incorporating new therapies.

Across clinical studies and real-world implementation analyses, subcutaneous (SC) immunotherapy reduced administration time by approximately 85–90% compared with IV infusion, frequently decreasing total treatment time from 30–60 min to less than 10 min [1, 10, 31–34, 37, 38, 42].

This reduction translated into meaningful operational gains, including reallocation of infusion chairs, improved nursing efficiency, and greater capacity for patients requiring complex intravenous therapies. Based on capacity modeling derived from current day hospital workflows, adoption of SC immunotherapy may enable up to a 40% reduction in oncology day hospital occupancy for eligible treatments, alleviating bottlenecks without requiring expansion of physical infrastructure [1, 10, 31–34, 37–39].

Beyond quantitative gains, shorter administration times improved workflow predictability, reduced scheduling variability, and enhanced staff satisfaction by allowing professionals to focus on higher-complexity clinical tasks rather than prolonged administration logistics [37, 42, 47–49].

#### Rationale for decentralized care delivery

The organizational benefits of SC immunotherapy extend beyond hospital efficiency and create the opportunity to redesign where treatment is delivered.

By simplifying administration logistics, SC delivery allows selected patients to receive treatment closer to home in community hospitals, ambulatory care centers, or structured outpatient facilities. However, decentralization should not be interpreted as universal transfer of care. Safe implementation requires strict patient selection and preserved specialist supervision.

Patients considered appropriate for decentralized SC administration were those with stable disease, good functional status (ECOG 0–1), no recent grade ≥ 2 immune-related adverse events, and no immediate need for complex radiological reassessment or therapeutic decision-making. In contrast, patients with active toxicity, clinical deterioration, poor performance status, or uncertain treatment indication should remain under direct hospital-based oncology supervision [1, 10, 31, 32, 37, 38, 54].

This distinction directly addresses one of the main concerns raised by stakeholders: decentralization must improve convenience without compromising clinical safety or delaying oncologist evaluation.

Importantly, patients with cancer receiving immunotherapy continue to require continuous medical oncology assessment, even during periods of radiological stability. Treatment indication, duration, toxicity interpretation, and progression management remain complex medical decisions that cannot be delegated solely to administration sites.

Therefore, decentralization represents redistribution of care delivery—not replacement of oncologist-led care.

#### Proposed hub-and-spoke care delivery model

Based on the integrated analysis of clinical evidence, organizational needs, and stakeholder input, a hub-and-spoke model was identified as the most appropriate framework for decentralized SC immunotherapy delivery.

In this model, the tertiary cancer center functions as the clinical hub, retaining responsibility for treatment indication, initiation, reassessment, and management of complex cases. Peripheral centers—including community hospitals and ambulatory facilities—act as spoke sites, where SC immunotherapy is administered to selected patients under standardized protocols and continuous specialist oversight [1, 10, 23, 40, 43–49].

The model is structured around five essential elements:

##### Centralized clinical decision-making

Treatment selection, initiation, radiological reassessment, treatment discontinuation, and major therapeutic modifications remain under the responsibility of medical oncologists at the hub.

##### Strict patient eligibility criteria

Only clinically stable patients fulfilling predefined eligibility criteria are candidates for decentralized administration.

##### Standardized toxicity monitoring

Uniform protocols for symptom assessment, laboratory surveillance, and immune-related adverse event recognition are applied across all care settings.

##### Defined escalation pathways

Rapid referral mechanisms to the hub are mandatory for patients with suspected immune-related toxicity, disease progression, or functional deterioration, in accordance with European Society for Medical Oncology guidance [54].

##### Training and accreditation

Healthcare professionals in spoke centers require specific training in SC immunotherapy administration, early recognition of immune-related toxicity, and escalation procedures [1, 10, 31, 32, 37, 38, 40, 43–49].

**Fig. 1:**
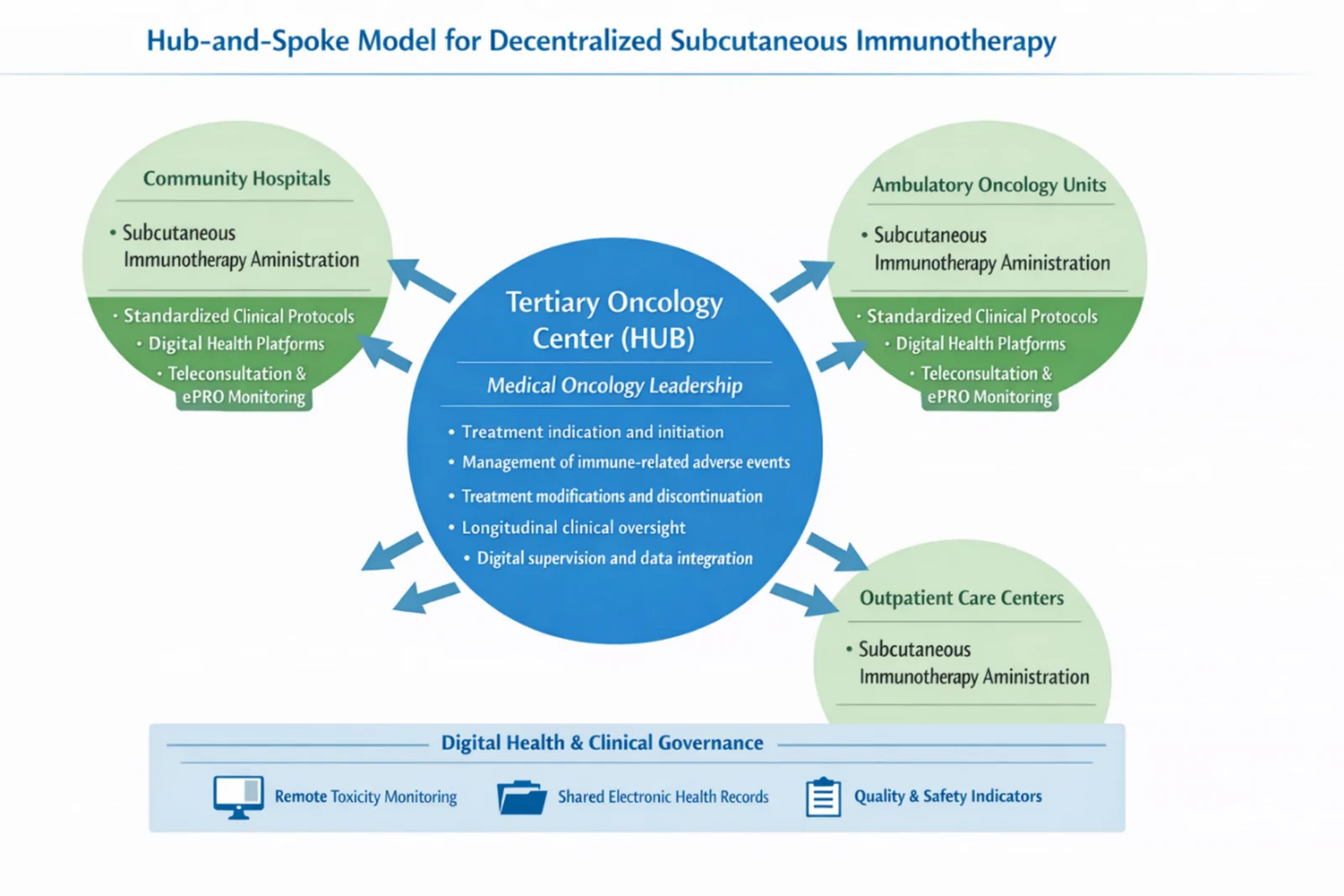
Proposed hub-and-spoke model for decentralized delivery of subcutaneous immunotherapy

This model explicitly prevents decentralization from becoming unsupervised outpatient administration or inappropriate task-shifting to non-specialist settings Fig. 1.

Clinical decision-making, treatment initiation, and toxicity management remain centralized within the tertiary oncology hub, led by medical oncology. Subcutaneous immunotherapy administration is delivered in community-based spoke centers under standardized protocols, supported by digital health tools, remote monitoring, and clearly defined escalation pathways to ensure safety, quality, and continuity of care [1, 10, 23, 31, 32, 37, 38, 40, 41, 43–45].

### Role of digital health and remote monitoring

Digital health tools were identified as a critical enabler of safe decentralization.

Remote monitoring of patient-reported outcomes, structured electronic symptom reporting, teleconsultations, and shared digital clinical records facilitate early detection of immune-related adverse events and maintain continuous oncologist supervision despite geographical distance from the tertiary center [1, 6, 10, 23, 36, 41, 44–46].

These tools are particularly relevant because immune-related toxicities may present subtly and require rapid specialist interpretation. Electronic monitoring improves early recognition and reduces the risk of delayed intervention.

Digital integration also reinforces patient confidence, as survey findings showed greater acceptance of decentralized administration when patients perceived preserved direct access to their treating oncologist and rapid re-escalation pathways.

Thus, digital health is not an optional complement, but a structural requirement for safe decentralized immunotherapy delivery.

### Preserving clinical coordination and quality of care

A major risk of decentralized oncology care is fragmentation of clinical responsibility.

This study found that both healthcare professionals and patients considered preservation of medical oncology leadership essential for safe implementation. While administration may occur outside tertiary centers, clinical accountability must remain centralized.

The proposed model therefore establishes a single clearly identified oncologist-led clinical responsibility for each patient. This avoids ambiguity regarding toxicity management, treatment continuation decisions, and progression assessment.

Decentralization should not imply de-medicalization, nor should it facilitate inappropriate independent prescribing by non-oncology specialties or unsupervised administration in settings lacking adequate governance.

Rather, medical oncology functions as the coordinating specialty, integrating multidisciplinary collaboration while preserving therapeutic coherence and patient safety [1, 10, 23, 31, 32, 37, 38, 41, 44–46].

This leadership model was consistently identified as a prerequisite for professional acceptance of decentralized SC immunotherapy pathways.

### Implications for healthcare professionals and organizations

Implementation of SC immunotherapy within a decentralized model has important implications for both healthcare professionals and healthcare organizations.

Reduced treatment time decreases pressure on oncology day hospitals, improves working conditions, and allows specialized staff to focus on complex oncological care rather than repetitive administration tasks. At the same time, decentralization requires investment in training, coordination, governance, and digital infrastructure.

From a system perspective, this model improves resource utilization without requiring expansion of hospital infrastructure. Rather than building larger day hospitals, healthcare systems may redesign care pathways using SC immunotherapy as an operational lever for more sustainable oncology delivery.

This approach aligns with broader value-based healthcare strategies by simultaneously improving patient experience, preserving specialist care quality, and enhancing long-term system sustainability [1, 10, 31, 32, 37–39, 47, 48].

**Fig. 2:**
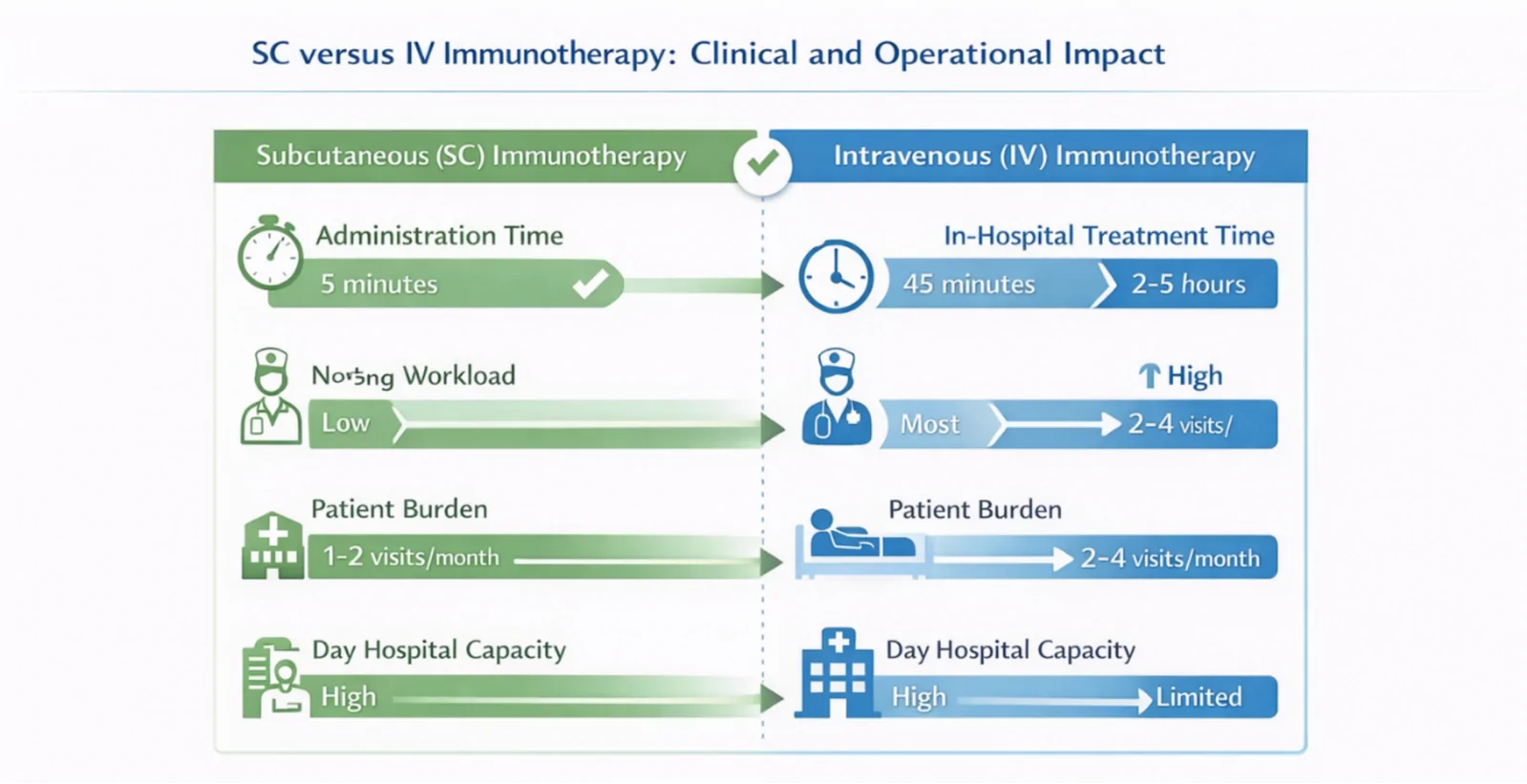
SC vs IV Immunotherapy: Clinical and Organizational Impact [1, 10, 31, 32, 37–39]

Figure. 2 Subcutaneous immunotherapy dramatically reduces administration and in-hospital treatment time, thereby decreasing nursing workload, lowering patient burden, and increasing day hospital capacity compared to intravenous administration. The figure illustrates relative differences in critical parameters using representative icons and scales for subcutaneous (SC) and intravenous (IV) immunotherapy [1, 10, 31, 32, 37–39, 42].

### Economic model and analytical perspective

The increasing use of immune checkpoint inhibitors has generated substantial pressure on the long-term sustainability of oncology care delivery. While immunotherapy provides major clinical benefit, its administration within conventional hospital-based models is associated with significant indirect costs related to nursing time, infusion infrastructure, day hospital occupancy, and opportunity costs derived from limited treatment capacity.

A comparative economic evaluation was performed from the perspective of the public healthcare system, focusing on administration-related differences between subcutaneous (SC) and intravenous (IV) immunotherapy.

A simplified decision-tree model was used to compare SC and IV administration pathways over a one-year time horizon. Because phase III studies demonstrated equivalent efficacy and survival outcomes between formulations, drug acquisition costs were assumed to be equivalent according to current pricing structures and were therefore excluded as a differentiating variable.

The model included the following cost components:

- nursing and administration time
- oncology day hospital chair occupancy
- pharmacy preparation and infusion infrastructure
- consumables associated with IV administration
- indirect patient-related costs associated with prolonged hospital attendance

This approach allowed evaluation of the economic value generated by logistical simplification rather than differences in antitumor efficacy [1, 10, 22, 31, 32, 37, 38, 48, 49].

### Cost-effectiveness analysis

Incremental cost-effectiveness ratios (ICERs) were estimated using quality-adjustd life years (QALYs) derived from published patient-reported outcome data and validated quality-of-life instruments, including EORTC QLQ-C30-based utility estimates.

Because no differences in progression-free survival or overall survival were observed between SC and IV formulations, incremental value was driven by improvements in treatment experience, reduced hospital time, and decreased treatment burden rather than survival gains.

Patients receiving SC immunotherapy showed modest but clinically meaningful improvements in utility values related to convenience, reduced disruption of daily life, and improved treatment satisfaction. When combined with lower administration-related costs, SC immunotherapy consistently remained below commonly accepted European willingness-to-pay thresholds, supporting a favorable cost-effectiveness profile [1, 6, 10, 22, 31, 32, 36–38, 46–48].

These findings reinforce that the value proposition of SC immunotherapy lies primarily in patient-centered outcomes and healthcare efficiency rather than superior oncological efficacy.

### Return on investment and institutional impact

A return on investment (ROI) analysis was performed to estimate the financial impact of SC immunotherapy adoption at institutional scale.

The base-case model incorporated reductions in administration time, nursing workload, oncology day hospital occupancy, and opportunity costs associated with infusion chair release. Under conservative assumptions, adoption of SC immunotherapy generated an estimated net annual saving of approximately €1,410 per treated patient.

Scenario analyses using higher adoption rates and extended treatment duration demonstrated even greater cumulative institutional savings, particularly in high-volume tertiary cancer centers where day hospital saturation is a major operational limitation [1, 10, 22, 31, 32, 37, 38].

These savings do not primarily reflect “cheaper treatment,” but rather more efficient use of existing hospital resources. Released capacity may be redirected toward patients requiring complex intravenous therapies, urgent oncological care, or new treatment indications without requiring expansion of physical infrastructure.

From a system perspective, this improves resilience and reduces the need for additional capital investment in oncology facilities [1, 10, 31, 32, 37, 38, 45–47].

### Sensitivity analyses and robustness of findings

Sensitivity analyses were performed using conservative and optimistic scenarios to test the robustness of the economic estimates.

Variables with the greatest impact included treatment volume, proportion of patients eligible for SC administration, local day hospital costs, and assumptions regarding staff time savings.

Even under conservative assumptions—including partial adoption rates and reduced operational efficiency gains—SC immunotherapy remained economically favorable compared with IV administration. Under optimistic scenarios involving broader implementation and extended-interval dosing strategies, economic benefit increased substantially.

These findings suggest that the economic advantage of SC immunotherapy is structurally robust and not dependent on highly optimistic assumptions.

However, economic benefit is strongly linked to appropriate implementation. Without protocolized patient selection, governance structures, and digital coordination, potential efficiency gains may be diluted or lost.

### Broader sustainability implications

Beyond direct institutional savings, SC immunotherapy supports broader sustainability goals aligned with value-based healthcare principles.

Reduced hospital visits decrease patient travel burden, time away from work, caregiver disruption, and indirect societal costs that are often underestimated in conventional hospital-based analyses. These benefits are particularly relevant for patients receiving prolonged immunotherapy over months or years.

Extended-interval dosing strategies (for example pembrolizumab every 6 weeks and nivolumab every 4 weeks), when combined with SC administration, further amplify these benefits by reducing both visit frequency and treatment duration.

This combination represents one of the most powerful operational strategies for improving sustainability in modern oncology day hospitals [27, 38, 46, 47, 51–53].

### Economic sustainability requires governance, not only efficiency

Importantly, economic savings should not be interpreted as justification for uncontrolled decentralization or reduction of specialist oversight.

Survey findings and benchmarking analyses consistently showed that safe implementation requires reinvestment of efficiency gains into training, digital infrastructure, toxicity monitoring systems, and coordination mechanisms between tertiary oncology hubs and peripheral spoke centers.

Subcutaneous immunotherapy improves logistical efficiency, but it does not reduce the clinical complexity of immunotherapy management. Patients continue to require specialized oncological assessment, longitudinal follow-up, and rapid access to expert management of immune-related adverse events.

Therefore, sustainable economic benefit depends on preserving strong medical oncology leadership rather than replacing specialist care with simplified administration pathways.

**Fig. 3:**
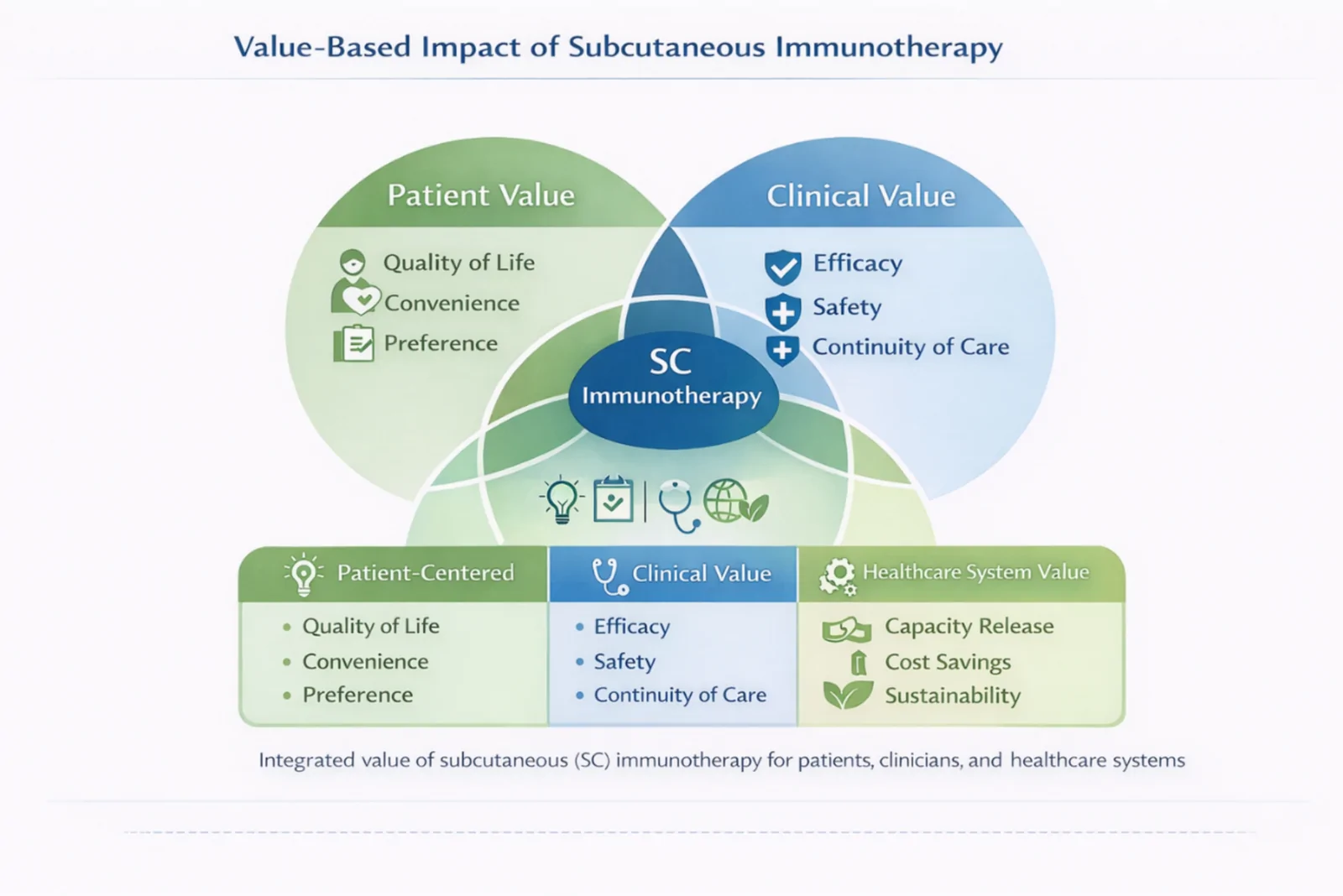
Value-Based Impact of SC Immunotherapy [1, 10, 31, 32, 37, 38, 46, 51–53]

The true value of SC immunotherapy lies in achieving efficiency without sacrificing safety, continuity, or quality of care Fig. 3.

Subcutaneous immunotherapy provides patient-centered, clinical, and system-level value by improving treatment experience, maintaining efficacy and safety, and optimizing healthcare resource utilization [1, 10, 31, 32, 37, 38]

## Results

### Stakeholder survey results

#### Survey population

A total of 160 participants were included in the survey analysis: 84 healthcare professionals and 76 patients receiving active immunotherapy treatment.

Healthcare professionals included medical oncologists, oncology nurses, hospital pharmacists, healthcare managers, primary care physicians, and pharmaceutical industry representatives involved in oncology care delivery. Patients were adults receiving immune checkpoint inhibitors for solid tumors in the tertiary care setting, including melanoma, non-small cell lung cancer, renal cell carcinoma, and other advanced malignancies.

The objective of the survey was to evaluate perceptions regarding subcutaneous (SC) immunotherapy, willingness to accept decentralized treatment delivery, perceived safety concerns, and the importance of maintaining oncologist-led care.

#### Healthcare professionals: perception of benefits and barriers

Among healthcare professionals, 89% considered SC immunotherapy to provide a clear organizational advantage compared with intravenous (IV) administration, primarily due to reduced administration time, improved day hospital capacity, and more efficient nursing resource allocation.

However, professional acceptance of decentralized administration was strongly dependent on governance conditions rather than route of administration alone. While 82% supported administration of SC immunotherapy outside tertiary hospitals for selected patients, only 18% considered broad decentralization appropriate without strict eligibility criteria and specialist supervision.

The most frequently reported concerns were:

- Delayed recognition of immune-related adverse events (74%)
- Fragmentation of clinical responsibility (68%)
- Loss of oncologist oversight (63%)
- Inappropriate treatment continuation without adequate reassessment (59%)

Medical oncologists and oncology nurses showed the strongest agreement regarding the need to preserve centralized clinical decision-making, particularly for treatment indication, radiological reassessment, and management of immune-related toxicity.

These findings reinforced that professionals viewed decentralization as a redistribution of care delivery rather than delegation of clinical responsibility.

#### Patients: preference for SC administration and care continuity

Among patients, 92% expressed preference for SC administration over IV treatment when efficacy and safety were considered equivalent.

The main reasons for preference included:

- Shorter time spent in hospital (91%)
- Reduced travel burden (76%)
- Greater flexibility for work and family life (69%)
- Lower perceived disruption of daily routines (64%)

However, patient preference was not based solely on convenience.

When asked about receiving treatment outside the tertiary oncology center, acceptance decreased substantially unless continued access to the treating oncologist was guaranteed. Only 34% of patients were comfortable with decentralized administration without regular specialist review, whereas 81% supported treatment closer to home when direct oncologist supervision, teleconsultation access, and rapid referral pathways were maintained.

Patients with longer treatment duration and stable disease showed greater acceptance of decentralized care, while those with recent toxicity, treatment changes, or disease progression preferred hospital-based administration.

This finding suggests that patients prioritize clinical security and continuity of care as much as convenience.

#### Perceived eligibility for decentralized administration

Both patients and professionals agreed that decentralization should be restricted to carefully selected clinical scenarios.

The profile most frequently considered appropriate for decentralized SC immunotherapy included:

- Stable disease
- ECOG performance status 0–1
- No recent grade ≥ 2 immune-related adverse events
- No need for immediate radiological reassessment
- Established treatment plan without imminent therapeutic modifications

Conversely, patients with clinical deterioration, uncertain treatment benefit, recent severe toxicity, or complex decision-making requirements were consistently considered inappropriate candidates for decentralized administration.

This alignment between patients and professionals strongly supported the selective hub-and-spoke model proposed in this study.

#### Importance of medical oncology leadership

Preservation of medical oncology leadership emerged as one of the strongest consensus findings across both stakeholder groups.

Among professionals, 94% considered oncologist-led governance essential for safe decentralized immunotherapy delivery. Similarly, patients identified continuity with a single responsible oncologist as a major determinant of trust and willingness to accept treatment outside the tertiary center.

Participants consistently distinguished between simplification of administration and simplification of cancer care, emphasizing that immunotherapy remains a complex treatment requiring continuous specialist assessment.

These findings support the principle that decentralization should enhance accessibility without weakening oncologist-led care coordination (53–56).

#### Implications for implementation

Survey findings demonstrated that acceptance of decentralized SC immunotherapy depends less on the subcutaneous route itself and more on the organizational model in which it is implemented.

Convenience alone was insufficient to justify decentralization. Safe adoption required:

- strict patient selection
- standardized toxicity monitoring
- clear escalation pathways
- digital communication tools
- preserved medical oncology leadership

This result provides strong real-world support for the proposed hub-and-spoke framework and directly reinforces the feasibility of decentralized, patient-centered oncology care when implemented under robust clinical governance.

## Benchmarking of existing decentralized oncology care models

### Selection of benchmark programs

A benchmarking analysis was conducted to identify existing national and international care models relevant to decentralized oncology delivery and to evaluate their applicability to subcutaneous (SC) immunotherapy.

Programs were selected based on their relevance to one or more of the following domains:

- hub-and-spoke oncology networks
- home-based or community-based systemic anticancer treatment
- shared-care oncology pathways
- digitally supported ambulatory oncology follow-up
- decentralized specialist cancer care with preserved tertiary center oversight

Representative initiatives from Spain, the United Kingdom, Australia, the Netherlands, India, and North America were included, encompassing both hospital-based and community-integrated oncology models [23, 40, 41, 43–45].

The objective was not comparative effectiveness ranking, but identification of transferable implementation principles and safety requirements relevant to SC immunotherapy delivery.

### Common organizational principles across successful models

Despite differences in healthcare systems and funding structures, successful decentralized oncology programs showed remarkable consistency in their core organizational principles.

The most recurrent elements were:

- preservation of centralized specialist decision-making
- strict patient selection criteria
- protocolized escalation pathways
- digital communication between peripheral and tertiary centers
- clearly assigned clinical responsibility
- formal training and accreditation of peripheral teams

Across all successful models, treatment decentralization involved redistribution of care tasks rather than transfer of oncological responsibility.

Tertiary centers retained responsibility for diagnosis, treatment indication, complex decision-making, and toxicity management, while peripheral sites focused on protocolized treatment delivery and early recognition of complications.

This structure strongly supports the proposed hub-and-spoke model for SC immunotherapy.

### National experiences

#### Spain and European models

Several Spanish and European experiences demonstrated the feasibility of partial oncology decentralization through coordinated hospital-community networks.

Programs such as home oncology initiatives, satellite day hospitals, and structured ambulatory treatment pathways showed improvements in accessibility and patient satisfaction without compromising clinical safety when clear escalation pathways were maintained.

In Spain, decentralized administration of selected supportive therapies and anticancer treatments has shown particular value in reducing travel burden for patients living far from tertiary referral centers. Similar experiences in the United Kingdom and the Netherlands have reinforced the role of shared-care pathways and digitally supported follow-up for stable patients receiving long-term treatment.

However, these programs consistently excluded patients with unstable disease, high toxicity risk, or complex decision-making needs, reinforcing the principle of selective rather than universal decentralization [23, 41, 44–48].

### International experiences

#### Australia, India, and home-based oncology programs

International models provided additional evidence regarding large-scale implementation of hub-and-spoke cancer care.

The Australian Oncology Shared Care Programme and NHS Cancer Home Care initiatives demonstrated that systemic anticancer treatment may be safely delivered outside tertiary hospitals when supported by formal governance, oncology leadership, and robust monitoring systems.

Similarly, technology-driven distributed oncology models implemented in India showed that digital integration and protocol standardization are critical to maintaining quality across geographically dispersed networks, particularly in high-volume systems with limited tertiary care capacity [43].

These models also highlighted that decentralization without strong governance leads to fragmentation, duplication, and delayed toxicity recognition. Successful implementation depended more on coordination quality than on the physical location of administration.

This finding is highly relevant for SC immunotherapy, where logistical simplification must not be confused with clinical simplification.

### Lessons from non-oncology biologics and subcutaneous therapies

Additional lessons were identified from the implementation of subcutaneous biologics in other specialties, particularly rheumatology and breast oncology.

The transition from intravenous to SC trastuzumab and daratumumab demonstrated substantial improvements in operational efficiency, patient preference, and day hospital capacity release. Similar findings were observed in long-term rheumatological biologic therapies administered through homecare pathways.

These experiences support the concept that route-of-administration change can drive major organizational transformation when supported by specialist oversight and patient education [28, 31, 32, 35, 50–53, 55, 56].

Although immunotherapy requires greater toxicity vigilance than many non-oncology biologics, these models provide important implementation lessons regarding training, monitoring, and patient empowerment.

### Barriers and limitations identified across benchmark programs

Benchmarking also identified recurrent implementation barriers.

The most frequent challenges included:

- uncertainty regarding clinical responsibility
- insufficient digital interoperability
- limited training of peripheral teams
- reimbursement models poorly aligned with decentralized care
- professional resistance related to safety concerns
- patient anxiety regarding reduced hospital specialist contact

These barriers were particularly relevant when decentralization was perceived as transfer of responsibility rather than coordinated care redesign.

Programs with unclear governance structures showed poorer adoption and lower professional confidence, emphasizing that successful decentralization requires organizational redesign rather than simple relocation of treatment delivery.

### Implications for the proposed SC immunotherapy model

Benchmarking findings strongly support the feasibility of decentralized SC immunotherapy when implemented through a selective hub-and-spoke framework.

No successful model supported independent administration without specialist oversight. Instead, all sustainable programs relied on preserved tertiary oncology leadership, strict eligibility criteria, digital monitoring, and rapid escalation pathways.

This evidence reinforces the central premise of the present study: subcutaneous immunotherapy should not be viewed merely as a more convenient administration route, but as an opportunity to redesign oncology care delivery while preserving safety, accountability, and oncologist-led coordination.

The proposed model therefore aligns with real-world implementation evidence and reflects organizational principles already validated across multiple healthcare systems.

## Discussion

This mixed-methods analysis supports subcutaneous (SC) immunotherapy as more than an alternative route of administration; rather, it represents a potential structural enabler for redesigning oncology care delivery. By combining clinical evidence, economic evaluation, stakeholder perspectives, and benchmarking of decentralized care models, our study suggests that SC immunotherapy may facilitate a transition toward more patient-centered, efficient, and sustainable oncology pathways without compromising safety or clinical quality.

A central finding of this work is that the value of SC immunotherapy does not arise from superior antitumor efficacy. Across pivotal studies, SC formulations of pembrolizumab, nivolumab, and atezolizumab demonstrated pharmacokinetic bioequivalence, comparable efficacy outcomes, and similar safety profiles compared with intravenous (IV) administration. The principal advantage therefore lies in logistical simplification: shorter administration times, reduced hospital attendance, and improved patient experience. This distinction is clinically important because it reframes SC immunotherapy not as a therapeutic escalation, but as an organizational opportunity for system redesign [1, 7, 24–26, 31, 32, 37, 38].

This is particularly relevant in the current oncology landscape, where immune checkpoint inhibitors have transformed several advanced cancers into chronic disease trajectories requiring prolonged treatment and repeated hospital contact. As immunotherapy indications continue to expand, tertiary oncology day hospitals face increasing saturation. Our findings confirm that reducing administration time by up to 85–90% may substantially improve operational efficiency, release infusion capacity, and reduce workforce pressure without requiring expansion of hospital infrastructure. In this context, SC immunotherapy becomes not merely a convenience innovation, but a strategic response to system-level sustainability challenges [31, 32, 37, 47, 55, 56].

However, one of the most important messages of this study is that decentralization should not be interpreted as de-medicalization. Patients receiving immunotherapy continue to require complex therapeutic decision-making, longitudinal reassessment, and rapid recognition of immune-related adverse events. Even in periods of radiological stability, decisions regarding treatment continuation, duration, and escalation remain specialist responsibilities. For this reason, our findings strongly support selective decentralization rather than universal transfer of care.

Only carefully selected patients—those with stable disease, good functional status, no recent significant immune-related toxicity, and no immediate need for complex reassessment—should be considered candidates for decentralized SC administration. This directly addresses one of the major concerns raised by both healthcare professionals and patients: convenience cannot justify loss of oncologist oversight. The concept of “treatment closer to home” is acceptable only when continuity of specialist-led care is preserved.

The proposed hub-and-spoke model provides a pragmatic framework to operationalize this balance. In this model, tertiary oncology centers retain responsibility for treatment indication, reassessment, and management of complex cases, while peripheral centers administer SC immunotherapy under standardized protocols and rapid escalation pathways. Importantly, this model is not theoretical. Benchmarking of national and international experiences demonstrated that successful decentralized oncology programs consistently rely on centralized governance, strict eligibility criteria, digital monitoring, and clearly assigned clinical responsibility. No sustainable model supported independent administration without specialist oversight.

This finding is particularly relevant in relation to concerns regarding the potential dilution of medical oncology leadership. Our study strongly supports that preserving oncologist-led governance is not professional protectionism, but a patient safety requirement. Immunotherapy toxicity is distinct from conventional chemotherapy toxicity, often unpredictable, and may require rapid multidisciplinary intervention. Delegating administration does not justify delegating clinical accountability. The distinction between drug delivery and therapeutic responsibility must remain explicit in any decentralized model.

The economic analysis reinforces this perspective. Because drug acquisition costs remain essentially equivalent, the economic benefit of SC immunotherapy derives from improved resource utilization rather than cheaper treatment. The estimated annual net saving of approximately €1,410 per patient reflects released chair capacity, reduced nursing time, and improved operational flexibility. When combined with extended-interval dosing strategies such as pembrolizumab every 6 weeks or nivolumab every 4 weeks, these gains may become even more substantial. However, economic value should not be interpreted as justification for uncontrolled decentralization. Savings must be reinvested into digital infrastructure, staff training, governance mechanisms, and toxicity monitoring systems to ensure that efficiency gains translate into safe implementation.

The stakeholder surveys add important nuance to this discussion. While preference for SC administration was expected, both patients and professionals clearly differentiated between convenience and safety. Patients valued reduced hospital time, but acceptance of decentralized care depended strongly on preserved access to their treating oncologist. Similarly, healthcare professionals supported decentralized administration only under conditions of strict patient selection and specialist-led governance. These findings strengthen the argument that implementation success depends more on organizational design than on the route of administration itself.

This study has several limitations. First, the literature review was structured but not designed as a formal systematic review or meta-analysis, which may limit completeness despite efforts to ensure methodological transparency. Second, the economic analysis was based on decision modeling and published operational estimates rather than prospective real-world cost comparisons. Third, survey findings may be influenced by selection bias and local organizational context, particularly given the tertiary-center setting. Finally, benchmarking analyses focused on transferable organizational principles rather than formal comparative effectiveness assessment. Nevertheless, the convergence of evidence across multiple independent domains strengthens the credibility and practical relevance of the proposed model.

Future research should focus on prospective implementation studies evaluating decentralized SC immunotherapy pathways in routine clinical practice. Real-world evidence regarding long-term safety, patient-reported outcomes, toxicity management, and cost sustainability will be essential. In addition, health system research examining reimbursement models, interoperability of digital platforms, and workforce adaptation across different healthcare settings will be necessary to support broader adoption.

Ultimately, SC immunotherapy should not be viewed simply as a faster way to administer treatment, but as an opportunity to redesign oncology care around the principles of value, accessibility, and continuity. The challenge is not whether decentralization is possible, but how to achieve it without weakening the specialist structures that ensure quality and safety.

## Conclusions

Subcutaneous immunotherapy represents a clinically effective and safe alternative to intravenous administration, but its greatest value extends beyond simplification of drug delivery. By maintaining equivalent efficacy and safety while substantially reducing treatment time and hospital resource utilization, SC immunotherapy creates a unique opportunity to redesign oncology care delivery in a more patient-centered and sustainable way.

The findings of this mixed-methods analysis suggest that the true impact of SC immunotherapy lies in its capacity to support selective decentralization of cancer care rather than universal transfer of treatment outside tertiary centers. Patients receiving immunotherapy continue to require specialized oncological assessment, longitudinal follow-up, and rapid management of immune-related adverse events. Therefore, decentralization must be understood as redistribution of care delivery—not de-medicalization of cancer treatment.

Safe implementation requires a structured hub-and-spoke model in which medical oncology retains clinical leadership over treatment indication, reassessment, and toxicity management, while selected patients may receive SC administration closer to home under standardized protocols, digital monitoring, and clearly defined escalation pathways.

Economic benefits derived from reduced administration burden and released oncology day hospital capacity further reinforce the sustainability of this model, but these gains should be reinvested into governance, training, and digital infrastructure to preserve quality and safety.

Ultimately, subcutaneous immunotherapy should be viewed not simply as a more convenient formulation, but as a strategic enabler of modern oncology care transformation. When implemented within robust oncologist-led governance frameworks, it offers the possibility of improving patient experience, optimizing healthcare resources, and strengthening long-term system sustainability in the immunotherapy era.

## Data Availability

not applicable
